# From Big Data to Artificial Intelligence: chemoinformatics meets new challenges

**DOI:** 10.1186/s13321-020-00475-y

**Published:** 2020-12-18

**Authors:** Igor V. Tetko, Ola Engkvist

**Affiliations:** 1 Helmholtz Zentrum München-German Research Center for Environmental Health (GmbH), Institute of Structural Biology, Ingolstädter Landstraße 1, 85764 Neuherberg, Germany; 2 BIGCHEM GmbH, Valerystr. 49, 85716 Unterschleißheim, Germany; 3 Molecular AI, Discovery Sciences, R&D, AstraZeneca, Gothenburg, Sweden

## Abstract

**Abstract:**

The increasing volume of biomedical data in chemistry and life sciences requires development of new methods and approaches for their analysis. Artificial Intelligence and machine learning, especially neural networks, are increasingly used in the chemical industry, in particular with respect to Big Data. This editorial highlights the main results presented during the special session of the International Conference on Neural Networks organized by “Big Data in Chemistry” project and draws perspectives on the future progress of the field.

****Graphical Abstract**:**

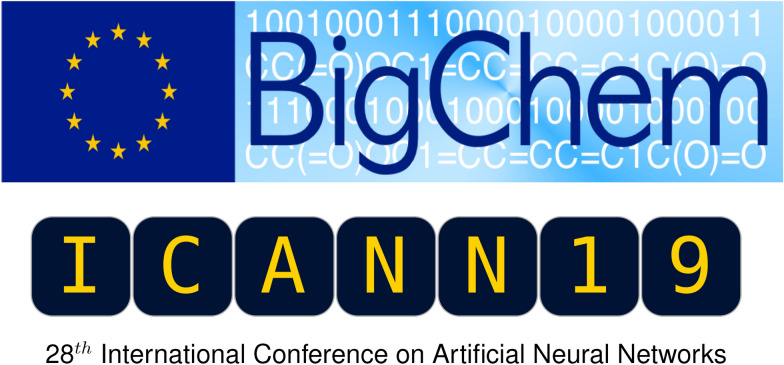

The analysis and exploitation of Big Data was the cornerstone of the “Big Data in Chemistry” (BIGCHEM), and of this special issue, which was prepared following the International Conference on Neural Networks (ICANN2019). In total 17 articles, including 15 contributions co-authored by BIGCHEM PhD students and partners, were published in this issue. Its thematic covered many different aspects of the use of Big Data in medicinal chemistry [1, 2] that were actively pursued and advanced during the project. The articles in the issue can be categorized into two main groups.

The first group deals with machine learning methods to improve analysis of large datasets such as those of high-throughput screening (HTS) campaigns. The comparison of structure-based and protein–ligand interaction fingerprints (IFPs) and for the prediction of ligand binding modes for protein kinases were studied by Rodríguez-Pérez et al. [3]. The authors showed that including target-relevant information via IPFs improved predictions of the modes by about 10% compared to the use of traditional atom environment fingerprints. Laufkötter et al. [4] demonstrated that augmenting chemical structure descriptors with bio-activity based fingerprints derived from HTS data provides better performance but, importantly, also superior scaffold hopping capability. Analogously QSAR-derived affinity fingerprints (QAFFP) [5, 6] outperformed classical Morgan fingerprints for scaffold hopping. While Morgan fingerprints due to their robustness and performance for small molecules (see review of David et al. [7]) are frequently used as a gold standard in, e.g., virtual screening and target predictions, they might not be optimal for larger molecules, such as peptides. MinHashed Atom-Pair fingerprints with a diameter of up to four bonds (MAP4) [8] were introduced as a universal fingerprint providing good results for various targets. HTS data are frequently imbalanced with only few active compounds: COVER (conformational oversampling as data augmentation for molecules) generates multiple conformations of molecules, in order to provide an efficient data balancing mechanism for the underrepresented class [9]. All these methodological studies are important to have better models for Big Data.

The second group of articles deals with novel machine-learning algorithms such as the use of generative models (GMs) for molecular *de novo* design in drug discovery. BIGCHEM was one of the originators in this area of research with its pioneering works on applying Recurrent Neural Networks (RNN) with reinforcement learning and variational autoencoders for molecular designs [10, 11] as reviewed elsewhere [12]. LatentGAN represents one of the most advanced developments of GMs by combining an autoencoder and a generative adversarial neural network [13]. The overfitting can reduce the diversity of autoencoder-generated structures. Generative Examination Networks (GEN) use randomized SMILES and early stopping [14] to prevent this [15]. The effect of the randomized SMILES to improve the quality of GMs is also confirmed with extensive benchmarking based on GDB-13 [16]. Another method to increase the diversity of generated structures was proposed by Blaschke et al. [17] who use memory-assisted reinforcement learning for this purpose. The methods developed in these studies are general ones and can be used to enhance other GMs such as scaffold decoration [18] or Mol-CycleGAN [19]. New types of deep learning algorithms based on Message Passing Neural Networks [20] and Transformer Convolutional Neural Networks (CNN) [21] were also introduced.

There is a significant difference between both groups of articles. The methods used in the first group mainly explore traditional machine learning methods, such as Random Forest, Support Vector Machines, etc. that are based on traditional molecular representations as a vector of descriptors [7]. Those studies could be performed using traditional toolkits with no or little programming effort. Contrary to that, the generative models were based on novel machine deep learning architectures such as CNN, RNN, Long Short Term Memory, Transformers, etc. These methods are more innovative and most of them were introduced, developed and/or implemented by the authors. All of the studies from this second group thus required significant programming skills and expertise in modern toolkits such as TensorFlow, Keras, Pytorch, etc., which are becoming a pre-requisite to get a position in the industry in addition, of course, to excellent knowledge in the basis disciplines. Prospective PhD students, who plan to build their careers in the field of chemoinformatics and Artificial Intelligence (AI), should not overlook these requirements.

One of the major differences of these new methods is their ability to infer statistical dependencies directly from chemical structures, which can be represented as text, e.g. SMILES [21, 22], chemical graphs [20], or 3D images [23]. The benchmarking studies [20, 22] show that such methods can achieve similar or better performances compared to traditional methods for classical tasks such as QSAR[24] but at the same time allow for intuitive interpretation of models [21]. Moreover, they can be used to address very different tasks, such as the aforementioned generation of molecules with desired properties or/and the prediction of single step (retro) synthesis [25, 26], or even complete retro-synthesis [27, 28] that could not be achieved with traditional methods. All these approaches are part of the emerging area of AI, which is going to drive the future of chemoinformatics.

AI is fast becoming a ubiquitous part of modern life, and is also increasingly employed in the pharmaceutical industry to automate key steps in drug design. Compared to Big Data challenges, “how to best analyse the Big Data” [1, 2], the future progress in this field is linked to the need for explainable “chemistry aware” methods. Such method should allow the elucidation of the molecular basis of compound activity, or to directly suggest new compounds with improved properties, or to optimise routes for synthesis supported with chemical knowledge. These and other topics, such as interpretable deep learning, use of knowledge elicitation from human experts, machine learning and molecular dynamics, language and quantum chemistry based retro-synthesis prediction, scalable multi-objective synthesis route optimization, methods for scaffold hopping, uncertainty estimation of AI methods, etc., will be investigated within the “Advanced machine learning for Innovative Drug Discovery” (AIDD, http://ai-dd.eu). This project will employ 16 fellows starting January 2021, who will get training and full support in theoretical and practical skills from their supervisors and via various network activities. While not being a direct continuation of BIGCHEM, AIDD will definitely contribute to the further development of the successful methods originated from the previous network.

The advance in this field critically depends on the availability of open source software, which is important for sustainable progress and sharing results. A distinct feature of BIGCHEM was the voluntary decision of its several partners to release the source code for its methodological developments, which dramatically boosted the respective research areas. For example the publicly available source code [29] from the REINVENT [10] article was forked 75 times since its publication, which definitely contributed to a rigorous validation, and a wide acceptance of the published results by the scientific community. The same principle will be widened in the AIDD, where all partners have agreed to release the source codes of their individual projects to improve their dissemination.

In summary, this special issue comprises a carefully selected collection of articles in Big Data, most of which were contributed by BIGCHEM partners or reported during the ICANN19 (http://e-nns.org/icann2019). Considering the great success of the project, which contributed about 70 publications that were cited nearly 1000 times in 2020 alone (https://scholar.google.com/citations?user=eLncF6MAAAAJ) as well as of the ICANN19, which was attended by all-time record of 500 participants and resulted in five volumes of proceedings [30], we believe that this special issue will be of a great interest to the readers of the journal.
